# EAST MEETS WEST: WORKING TOGETHER TOWARD AN AGE-INCLUSIVE SOCIETY THROUGH RESEARCH ON AGEISM

**DOI:** 10.1093/geroni/igae098.1016

**Published:** 2024-12-31

**Authors:** Takashi Yamashita, Limei Chen, Nancy Kusmaul

**Affiliations:** Chair; Co-Chair; Discussant

## Abstract

This East-Meets-West symposium aims to address how ageism as age-based discrimination, prejudice, and stereotypes manifests across various settings in Japan and the United States. Ageism can lead to negative outcomes such as missed employment opportunities, poor health, and lower social well-being. This symposium will serve as a platform to integrate findings and insights from the ageism studies in two distinctive Eastern and Western cultural zones. Although both countries are economically developed nations and experiencing population aging, the perception and treatment of older adults as well as their strategies to cope with ageism significantly vary due to distinctive cultural norms, health care systems, and social support systems. This symposium consists of four presentations. Dr. Yasumoto will present findings on age stereotypes and longevity aspirations among older Japanese people. Dr. Takeuchi will present findings from a large-scale survey research and differential effects of ageism by gender and employment in Japan on older adult’s health and social participation. Dr. Gonzales will present findings from their analysis of the U.S. Health and Retirement Study on how ageism affects racial and ethnic minorities’ health and civic engagement. Finally, Dr. Mulhorn will present findings on the prevalence and situated use of ageist terms on Twitter based on 60 million tweets. The symposium will provide a discussion to triangulate findings from these four studies and to identify culturally sensitive innovative solutions to address ageism and break barriers for all individuals to achieve high quality of life at any age over the life course. Japanese and Japanese American Aging Studies Interest Group Sponsored Symposium

